# Inferior Mesenteric Arteriovenous Fistula Successfully Treated with Surgical Resection: A Case Report

**DOI:** 10.3400/avd.cr.25-00067

**Published:** 2025-10-11

**Authors:** Takuya Shimizu, Miho Kamakura, Yoshihisa Murata, Kazuhiro Ota, Miki Takeda, Wakiko Hiranuma, Takayuki Matsuoka, Tadanori Minagawa, Fukashi Serizawa, Masato Ohara, Yuko Itakura, Shunsuke Kawamoto

**Affiliations:** 1 Department of Cardiovascular Surgery, Tohoku Medical and Pharmaceutical University Graduate School of Medicine, Sendai, Miyagi, Japan; 2 Department of Surgery, Japanese Red Cross Ishinomaki Hospital, Ishinomaki, Miyagi, Japan; 3 Department of Pathology, Japanese Red Cross Ishinomaki Hospital, Ishinomaki, Miyagi, Japan

**Keywords:** inferior mesenteric artery, arteriovenous fistula, surgical resection

## Abstract

Idiopathic inferior mesenteric arteriovenous fistula is an extremely rare pathology, and symptoms vary greatly depending on the shunt flow volume through the fistula. We report a case of idiopathic inferior mesenteric arteriovenous fistula in a 63-year-old man who presented with a pulsating sensation in the upper abdomen. Computed tomography revealed an inferior mesenteric arteriovenous fistula with aneurysmal dilatation and a drainage vein into the dilated marginal vein of the descending colon. Surgical resection and ligation of the fistula were successfully performed, and the postoperative course was uneventful. The patient’s symptoms resolved, and no recurrence was observed during the 5-year follow-up.

## Introduction

Inferior mesenteric arteriovenous fistula (IMAVF) is an extremely rare pathology, with only 37 cases reported to date.^1–7)^ The presentation varies greatly depending on the blood flow volume through the fistula, ranging from minimal symptoms such as abdominal pain to severe ischemic colitis and gastrointestinal bleeding caused by portal hypertension.^1–4,7)^ Treatment options include surgical resection and catheter embolization; however, because of its rarity, clear guidelines for the treatment of IMAVF, especially with respect to its size, are lacking. Herein, we present a case of an IMAVF with aneurysmal dilatation that was successfully treated with resection and ligation of the fistula.

## Case Report

**Fig. 1:**
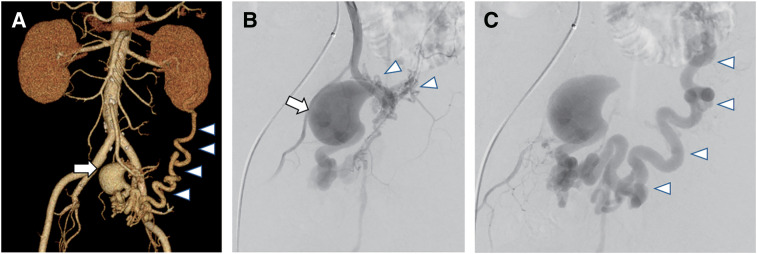
CT and angiography. (**A**) A contrast-enhanced CT shows an aneurysm-like dilatation of the IMAVF (arrow) and a tortuous drainage vein of the descending colon flowing into the splenic vein (arrowheads). (**B**, **C**) Selective angiography of the IMAVF shows a markedly dilated AVF (arrow) with a nidus supplied by multiple feeding arteries (arrowheads) originating from the inferior mesenteric artery in the early phase (**B**) and a drainage vein in the late phase (arrowheads) (**C**). AVF: arteriovenous fistula; CT: computed tomography; IMAVF: inferior mesenteric arteriovenous fistula

A 63-year-old man, who had been diagnosed with paroxysmal atrial fibrillation 8 years prior, presented to our hospital with insomnia caused by a pulsating sensation in the upper abdomen. The patient was otherwise in good health and had no history of abdominal surgery or trauma. Physical examination revealed no abnormal findings in the abdomen, and laboratory test results were unremarkable. Contrast-enhanced computed tomography (CT), performed based on a suspicion of abdominal aortic aneurysm, revealed a 3.0-cm aneurysmal dilatation of the sigmoid branch of the inferior mesenteric artery (IMA) and a dilated and tortuous single marginal vein of the sigmoid and descending colon in the arterial phase, suggestive of an IMAVF (**Fig. 1A**). IMA angiography revealed a dilated vessel and a fistulous nidus supplied by multiple feeding arteries from the sigmoid branch of the IMA (**Fig. 1B**). The large nidus drained into the splenic vein via an enlarged and tortuous marginal vein (**Fig. 1C**). Upper gastrointestinal endoscopy and colonoscopy revealed no significant findings. Based on these findings, the patient was diagnosed with IMAVF without colonic ischemia.

**Fig. 2:**
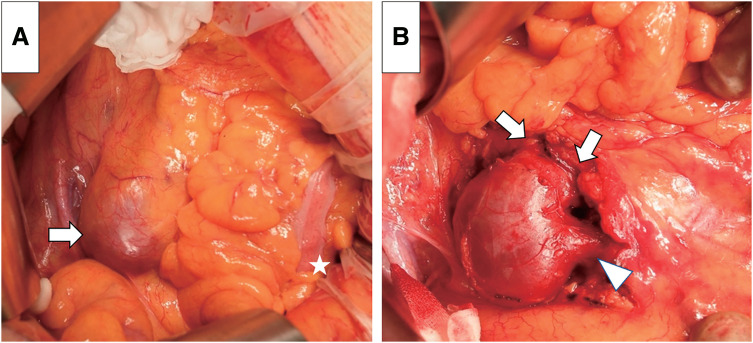
Operative findings. (**A**) The dilated IMAVF is partly buried in the sigmoid mesocolon (arrow). The white star indicates the sigmoid colon. (**B**) Multiple feeding arteries (arrows) and the main drainage vein (arrowhead) have been ligated, and the large IMAVF has been resected. IMAVF: inferior mesenteric arteriovenous fistula

Surgical resection of the dilated vessel and ligation of the multiple feeding arteries of the fistula via a median laparotomy was planned to eliminate the symptoms and prevent rupture of the dilated vessel. The dilated fistula was partly buried inside the sigmoid mesocolon (**Fig. 2**). It was considerably soft, and a prominent thrill was palpable over it. The fistula was dissected free from the surrounding tissue, its multiple feeding arteries and drainage vein were ligated, and the thrill was eliminated, with no ischemic findings in the sigmoid and descending colon.

**Fig. 3:**
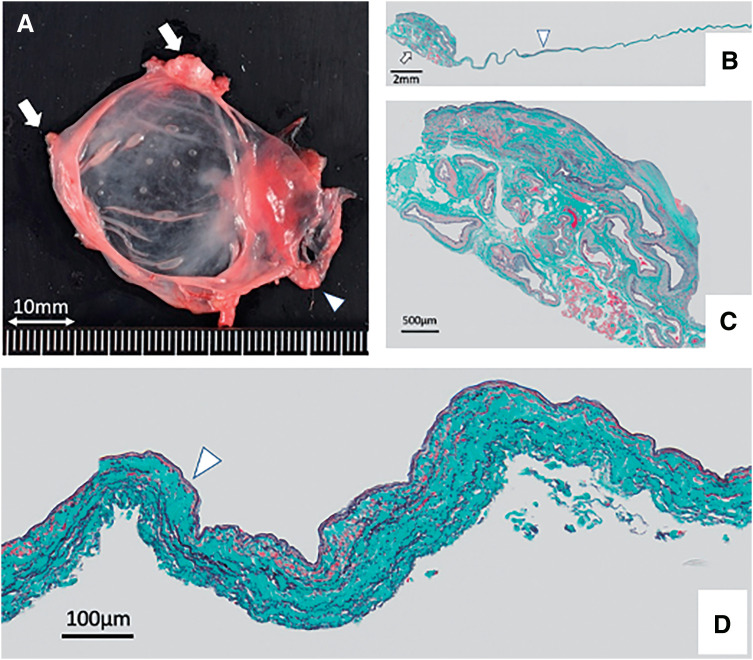
Pathological findings of the IMAVF. (**A**) Gross view of the IMAVF. The vessel wall of the dilated IMAVF is extremely thin. The arrows and arrowhead indicate the feeding arteries and drainage vein, respectively. (**B**) Low-power view. Elastica–Masson staining of the resected tissue shows multiple dilated vessels (arrow) and thinning of the enlarged aneurysmal vein of the IMAVF (arrowhead; scale bar: 2.0 mm). (**C**) High-power view shows closely packed multiple dilated vessels, consistent with an arteriovenous malformation (scale bar: 500 μm). (**D**) High-power view shows loss of smooth muscle cells (arrowhead) and scattered elastic fibers in the aneurysmal vein wall. The thickness of the enlarged aneurysmal vein wall is only 0.1 mm (scale bar: 100 μm). IMAVF: inferior mesenteric arteriovenous fistula

Pathological examination confirmed IMAVF with a wall thickness of only 0.1 mm. Furthermore, part of the dilated vessel wall had only multiple layers of collagen fibers without any elastic lamellae or smooth muscle cells (**Fig. 3**).

The postoperative course was uneventful, and the patient was discharged on postoperative day 8. The pulsatile sensation in the upper abdomen completely disappeared, and no recurrence was observed during the 5-year follow-up.

## Discussion

An arteriovenous fistula (AVF) decreases arterial blood flow to the organ distal to it via the steal phenomenon. Additionally, it increases venous pressure distal to the fistula. In patients with IMAVF, these 2 mechanisms exacerbate colonic ischemia in a synergistic manner, resulting in abdominal pain and lower intestinal bleeding with portal hypertension.

According to previous reports, >80% of patients with IMAVF have abdominal pain or lower gastrointestinal bleeding.^1)^ However, the clinical signs and symptoms of IMAVF vary depending on the blood flow volume in the fistula.

In the absence of specific symptoms that help in the diagnosis, a thorough examination is important to avoid missing IMAVF. A palpable dilated AVF or an enlarged colon due to ischemic enteritis, as well as an abdominal vascular murmur due to a high-flow AV fistula, have been reported in approximately 25% of IMAVF cases each.^1)^

Interestingly, the main symptom in this case was a pulsatile sensation in the upper abdomen. This suggests that the shunt flow volume was high even in the absence of symptoms associated with colonic ischemia and that rupture of the dilated vessel was imminent.

Similar to previously reported cases, our patient was in his early 60s and had no history of abdominal surgery or trauma; therefore, it is reasonable to assume that the IMAVF was idiopathic. CT was performed to detect a possible aneurysm formation in the abdomen. CT is useful for the diagnosis of IMAVF. Characteristic CT findings of IMAVF include dilated feeding arteries with dilated and tortuous drainage veins enhanced in the arterial phase, as well as abnormal thickening of the colon suggestive of colonic ischemia. Angiography is inevitable because it can provide precise information about the distribution of the fistula nidus and important clues regarding the management of the IMAVF.

Treatment options for IMAVF include surgical resection and transcatheter embolization. Catheter embolization is considered less invasive and relatively safer, but is associated with the risk of organ ischemia.^1)^ Some authors advocate that AVFs with a diameter >8 mm should not be treated with catheter embolization because of the risk of distal migration of the embolization material.^8)^ Furthermore, catheter embolization is associated with a risk of recurrence, especially if multiple feeding arteries are involved.

Of the 22 previously reported cases of idiopathic IMAVF, 12 (55%) were complicated by intestinal ischemia, and 9 (75%) of these required bowel resection. Of the 10 (45%) patients without intestinal ischemia, laparotomy was performed in 7 (70%), and intestinal resection was performed in 3 (43%). Only 2 (20%) of the 10 patients without intestinal ischemia underwent endovascular treatment.

A high percentage of patients without intestinal ischemia underwent bowel resection during IMAVF treatment. Bowel resection could be avoided in patients in whom the IMAVF was located proximal to the IMA and the marginal artery of the sigmoid colon could be spared. In other words, determining whether the marginal artery can be preserved based on the angiographic findings is important for assessing the feasibility of endovascular treatment for IMAVF. If the marginal artery can be preserved, the risk of intestinal ischemia is low, and endovascular treatment is indicated. Therefore, performing angiography is important when planning IMAVF treatment. If the feeding artery branching from the marginal artery alone is ligated and the aneurysm is resected via laparotomy, the marginal artery can be preserved and intestinal ischemia can be avoided.

In this case, angiography revealed an aneurysmal IMAVF branching directly from the peripheral side of the IMA trunk and multiple feeding arteries from the marginal artery. Therefore, endovascular treatment was contraindicated owing to the high risk of sigmoid colon ischemia caused by embolization.

In our patient, surgical resection was selected to treat the IMAVF because the lesion was not considered amenable to catheter embolization owing to the risk of colon ischemia or necrosis. Moreover, laparotomy provided some advantages over catheter intervention. We could directly confirm that the shunt flow was completely stopped without any ischemic changes in the colon during surgery. This suggests that resecting the IMAVF under direct visualization to confirm the absence of bowel ischemia is a reasonable treatment strategy.

Pathological examination revealed that the vessel-wall thickness was only 0.1 mm, and smooth muscle cells and collagen fibers were diminished in most areas, indicating a high risk of rupture. Although IMAVF rupture has not been reported, rupture of an aneurysmal coronary AVF approximately 30–40 mm in diameter has been reported.^9)^ Among venous aneurysms occurring in cerebral arteriovenous malformations, those with increased blood flow have a higher risk of rupture.^10)^

Therefore, it is reasonable to recommend that a dilated IMAVF with a diameter of at least 3 cm should be treated even in the absence of complications.

## Conclusion

IMAVFs are extremely rare, and their treatment remains controversial. CT and angiography are important for diagnosis and treatment planning. Surgical resection of dilated vessels and ligation of the feeding arteries and drainage veins without bowel resection are effective and safe options for the treatment of IMAVF without colon ischemia.
